# Poster Session I - A137 FROM REFERRAL TO RELIEF: IMPROVING WAIT TIMES FOR URGENT GASTROENTEROLOGY CONSULTATION IN CALGARY

**DOI:** 10.1093/jcag/gwaf042.137

**Published:** 2026-02-13

**Authors:** C M Ray, M Mazurek, E J Cheng, G Heather, C Ormond, W Sauve, S Heitman, M Brahmania

**Affiliations:** Gastroenterology, University of Calgary, Calgary, AB, Canada; Gastroenterology, University of Calgary, Calgary, AB, Canada; Gastroenterology, University of Calgary, Calgary, AB, Canada; Gastroenterology, University of Calgary, Calgary, AB, Canada; Gastroenterology, University of Calgary, Calgary, AB, Canada; Gastroenterology, University of Calgary, Calgary, AB, Canada; Gastroenterology, University of Calgary, Calgary, AB, Canada; Gastroenterology, University of Calgary, Calgary, AB, Canada

## Abstract

**Background:**

Prolonged wait times for gastroenterology (GI) specialist care is a longstanding issue in Canada, contributing to increased patient frustration, symptom burden and potential adverse outcomes. The *Canadian Association of Gastroenterology (CAG) Wait Time Consensus Group* created recommended guidelines for consultation and procedure wait times across 24 areas of gastroenterology according to acuity, which is used as a benchmark for Canadian institutions. Gastrointestinal diseases contribute to 15% of national health care expenditures. Currently, it is estimated that 1.8 gastroenterologist are needed per 100,000 people in Canada, and with rising rates of both international and interprovincial migration to Calgary, further improvement initiatives are needed to access to GI specialist services. Previous work in Calgary resulted in the creation of a GI central access triage (CAT) system, a single point entry model with a goal improving access to GI specialist care. While overall successful, the high demand for GI services continues to result in long waitlists.

**Aims:**

To reduce the median wait times for urgent GI consultations from 180 to 90 days, within 24 months, to better align with CAG Wait Time Consensus Group recommendations.

**Methods:**

Using the QI Model for Improvement framework, we outlined the current process for GI referrals through the CAT system. Data collection began in July 2025 and is continuing monthly through the first PDSA cycle. The planned interventions include waitlist clean up tools, workforce planning, and “three-strike” policy for unresponsive referrals. Our outcome measure is ‘days on the waitlist,’ the total time from referral to urgent consult appointment (target is 90 days). Process measures include number of duplicates or previously seen patients removed, and balancing measures include burden on nursing and physician teams.

**Results:**

In June and July 2025, GI CAT received 2423 and 2389 total consults respectively. The first PDSA cycle is currently underway. The baseline median wait time for urgent consults is 186 days. There were 1327 patients on the urgent waitlist, pre-initiative (June 2025), and data from July 2025 shows 1377 patients. By July 2025, less patients (77/1377) were waiting greater than 180 days compared to June 2025 (89/1327). Quantitative data on timeliness and completion rates will be analyzed following the first PDSA cycle.

**Conclusions:**

While the initial data does not demonstrate significant changes in total number of patients, initiative implementation began closer to mid-July, so these results may not accurately reflect the impact. We anticipate that implementation of iterative PDSA cycles will improve median wait times for access to urgent GI specialist care, with the goal of sustainability and minimizing staff fatigue to create an enduring impact.

**Funding Agencies:**

None

